# Perceived Effectiveness of Public Health Unit Partnerships With Faith-Based and Other Community-Based Organizations to Promote COVID-19 Vaccination Among Ethnoracial Communities

**DOI:** 10.3389/ijph.2024.1607200

**Published:** 2024-11-12

**Authors:** Melodie Yunju Song, Denessia Blake-Hepburn, Monali Varia, Elizabeth Estey Noad, Nazia Peer, Barry Pakes, Shaza A. Fadel, Sara Allin, Anushka Ataullahjan, Erica Di Ruggiero

**Affiliations:** ^1^ Dalla Lana School of Public Health, University of Toronto, Toronto, ON, Canada; ^2^ Region of Peel – Health Services, Mississauga, ON, Canada; ^3^ Department of Family and Community Medicine, Faculty of Medicine, University of Toronto, Toronto, ON, Canada; ^4^ Division of Clinical Public Health, Dalla Lana School of Public Health, University of Toronto, Toronto, ON, Canada; ^5^ Institute of Health Policy Management and Evaluation, Dalla Lana School of Public Health, University of Toronto, Toronto, ON, Canada; ^6^ School of Health Studies, Faculty of Health Sciences, Western University, London, ON, Canada; ^7^ Division of Social and Behavioural Health Sciences, Dalla Lana School of Public Health, University of Toronto, Toronto, ON, Canada

**Keywords:** community engagement, faith-based organizations, vaccine uptake, public health partnerships, ethnoracial communities

## Abstract

**Objectives:**

The objective of this study was to explore how Ontario Public Health Units (PHUs) partnered with faith-based organizations (FBOs) and other community-based organizations (CBOs) to promote COVID-19 vaccination among ethnoracial groups made structurally vulnerable during the pandemic, and to understand how PHUs perceive the effectiveness of these partnerships with these organizations.

**Methods:**

Between June to December 2022, we distributed a cross-sectional survey to 34 PHUs in Ontario to explore how PHUs were engaging and partnering with FBOs and CBOs.

**Results:**

Responses were received from 28 of 34 (82.5%) public health units. Across Ontario, 23 (82.1%) respondent PHUs worked with FBOs during the COVID-19 vaccine rollout with activities ranging from informing FBOs of vaccine availability, to using places of worship as sites for vaccine administration and co-creating educational materials on immunization that were faith- and culturally sensitive.

**Conclusion:**

FBOs can be a valuable community partner as PHUs work to increase the reach and uptake of public health interventions. Ongoing monitoring and evaluation of the impact of FBO engagement on vaccine confidence and uptake among ethnoracial communities is needed to inform future community engaged vaccine programming in Ontario.

## Introduction

Faith-based organizations (FBOs), defined as “entities dedicated to specific religious identities, often including a moral and social component” [1], are organizations that have historically collaborated with public health agencies to provide outreach and social services [2]. FBOs can, and have, supported public health agencies in addressing social and health inequities including improving equitable vaccine access during health emergencies [3, 4].

Faith-based communities are important participants in vaccine uptake since religion shapes individual and collective behaviours; thus, deliberate engagement with local faith actors and FBOs may beget social mobilization and vaccine awareness [5–7]. FBOs function as influential informal institutions that provide social capital, and ensure the health and wellbeing of the communities they serve [8]. A systematic review from the early stages of the COVID-19 pandemic, showed that faith-based communities contributed to reduced COVID-19 transmission by adapting and promoting practices that adhered to public health guidelines [9].

When COVID-19 vaccines became available, faith-based and community partnerships were employed as a strategy to co-create and implement COVID-19 vaccine awareness interventions to reach groups made structurally vulnerable [10]. Structural vulnerability refers to “an individual’s or a population groups’ condition of being at risk for negative health outcomes through their interface with socioeconomic, political and cultural/normative hierarchies” [11].

Ethno-racial groups made structurally vulnerable (e.g., Black, South Asian, Latinx) were disproportionately impacted by COVID-19 due to historical and structural determinants such as systemic racism, and mistrust in government organizations including the health sector [12–14]. The 2020 General Social Survey on Social Identity revealed that the main racialized groups in Canada: South Asian, Chinese, Black, Filipino, Arab, Latinx, Southeast Asian, West Asian, Korean and Japanese populations were more likely to report having experienced discrimination during the pandemic [15]. Many of these groups had lower than average vaccination rates, though disparities among these groups were observed [16–19]. For example, many South Asians were disproportionately affected in Peel region (Ontario) due to many residing in multi-generational homes and overrepresentation in essential work that could not be done remotely [20]. However, once COVID-19 vaccines were available, many of these individuals sought to be vaccinated and the disparity in COVID incidence declined which wasn't necessarily seen in other racialized groups [17].

To improve their reach to ethno-racial groups made structurally vulnerable, public health institutions turned to FBOs to promote vaccines in areas where demographic composition consisted of a diverse array of religious and ethnic representation. Church-based programs for Latin communities [21], Black communities [22]; as well as mosque-based programs for Muslim communities [23] were strongholds to improve vaccine awareness and uptake. Given the absence of a mandate to collect and report race-based data, PHUs used community engagement strategies (i.e., engagement with FBOs) to involve these groups [10]. While this was evident in the Canadian context, public health and FBOs have not always aligned on public health measures. Pushback from religious movements like Protestant churches and organizations regarding religious gatherings during the pandemic were apparent [24].

In Canada, the Public Health Agency of Canada (PHAC) and the National Advisory Committee on Immunization (NACI) undertook an extensive consultation program to design an equitable approach to prioritize COVID-19 vaccines [25, 26]. The NACI made recommendations for public health program level decision-making to increase access to immunization services to reduce health inequalities and engage systemically marginalized and racialized populations, disproportionately impacted by the pandemic, in immunization program planning [27]. Ontario PHU guidance on priority groups (e.g., health workers, people in congregate settings and adults in First Nations, Metis and Inuit populations) came from the Ministry of Health and the Ministry of the Solicitor General who adapted from the NACI recommendations [28]. National and provincial guidance informed the work of local PHUs which aimed to prioritize those most vulnerable in their communities [29, 30].

Considering the diverse populations served, PHUs designed and implemented localized approaches to execute vaccine distribution plans [31, 32]. These efforts were often supplemented by additional financial support from the federal and provincial government. For instance, PHAC funneled 45.5 million dollars over 2.5 years, through the Immunization Partnership Fund, to sponsor community-based organizations (CBOs) to raise vaccine awareness and close the rate gap among communities with low vaccination rates [33]. In Ontario, the provincial government launched the “High Priority Communities Strategy” in December 2020 and provided funding to lead community agencies and community partners residing within 15 priority neighborhoods disproportionately affected by COVID-19, in Durham, Peel, Toronto, York, and Ottawa [34, 35]. These priority communities were determined based on “high COVID-19 prevalence, low testing rates, and sociodemographic barriers to testing and self-isolation” [35].

Given the significant investments in community-centred initiatives to address inequities in vaccine uptake, it is important to document and evaluate these approaches. The objective of this study was to explore how PHUs partnered with FBOs and other CBOs to promote COVID-19 vaccination among ethnoracial groups made structurally vulnerable, and to understand how PHUs perceive the effectiveness of their partnerships with FBOs and other CBOs to implement COVID-19 vaccine interventions in Ontario. While ethno-racial groups made structurally vulnerable are not the sole beneficiaries of FBOs, we were specifically interested in investigating how PHUs collaborated with FBOs to reach these groups, given that many of these groups may also have faith affiliations [15]. Terms such as “minorities,” “equity-deserving,” “racialized” are widely used in the literature and evident in survey and PHU responses, however we use the term “ethnoracial groups made structurally vulnerable” in this paper to acknowledge the structural determinants (e.g., systemic racism) which impede access to healthcare and shape the healthy choices (e.g., vaccine decisions) of ethnoracial groups that experience structural vulnerability [11].

## Methods

This project was granted ethics approval by the University of Toronto (#42490).

### Study Design and Setting

In Ontario, there are 34 PHUs that deliver public health programs and services in accordance with the Ontario Public Health Standards (OPHS) [36]. We conducted a cross-sectional survey containing close-ended and open-ended questions with PHUs in Ontario from 1 June to 22 December 2022.

### Cross-Sectional Survey Design

To develop the survey, we first referred to several frameworks used to evaluate faith-based engagement in the context of immunization program delivery for ethnoracial communities made structurally vulnerable [4, 37, 38]. We chose the Consolidated Framework for Implementation Research (CFIR), a well-accepted and frequently used framework in implementation studies [39]. Survey questions were designed based on five conceptual domains to collect data on the interventions (domain 1), inner (domain 2) and outer setting (domain 3), roles and characteristics of interventions and respondents (domain 4) and implementation processes (domain 5) used by PHUs to partner with FBOs and other CBOs to improve vaccine confidence and uptake among ethnoracial communities made structurally vulnerable [39].

To capture *with whom, how, and by what means are PHUs working with FBOs, and what evaluation methods were involved*, we developed a 4-Part 24 question electronic survey (See “Survey Questions” in Supplementary Material S1). The survey was developed by MYS and validated and tested by the core research team (DBH, EDR, SA, AA, SAF). Respondents could also directly upload documents which included resources and materials created with FBO partnerships. Finally, PHUs were invited to send relevant documents and multimedia resources via email to further demonstrate how they worked with FBOs to encourage vaccine uptake among priority ethnoracial groups. Our survey covered all PHUs involved in the High Priority Communities Strategy. The survey captured PHU assessment of their vaccine programs’ incorporation of antiracism principles and gender considerations; the degree of involvement of FBOs in the design, implementation, and evaluation of the vaccine program; and description of formal and informal methods that PHUs use to evaluate the success of faith-based interventions for reaching priority ethnoracial groups. Anti-racism principles include: (i.) broad activities that account for the inherent power inequities in vaccine delivery programs (e.g., weighting staffing needs to distribute vaccinations based on race-based infection data); (ii.) dedicating resources to the design and delivery of vaccine programs that address unique race-and-gender based needs; (iii.) meaningfully involving ethnoracial communities made structurally vulnerable in the decision-making process; (iv.) taking positive action to incrementally redress racial inequities (e.g., introducing Public Health Standards that introduce racially concordant community-led vaccine promotion specialists in communities made vulnerable); and (v.) measuring progress of vaccine interventions in advancing racial equity [40].

To increase survey response rate, a Tailored Design Method (TDM) was applied [41] to ensure easily comprehensible language and concise instructions and clarifications were used throughout, such as graphs to support the understanding of the question. We showed respondents their progress in percentages when answering questionnaires and applied conditional formatting for numeric or text responses where needed to reduce response errors. Participants were given the option to leave their survey and return later to reduce incomplete survey responses.

### Recruitment and Survey Administration

We acquired a list of contacts responsible for overseeing COVID-19 immunization delivery in 34 respective PHUs from the Ontario Ministry of Health in May 2022. Using purposeful sampling, we distributed the survey to a total of 84 people, including 18 Medical Officers of Health, 43 immunization program specialists, 12 emergency response managers, and 11 relevant program delivery personnel within PHUs. We applied a hierarchical approach, first sending the survey to immunization program specialists and emergency response managers listed as first lines of contact.

We used REDCap Survey Distribution Tools to distribute the questionnaire via REDCap (Research Electronic Data Capture) and to keep track of response status. We sent out 3 to 5 reminder emails, 2 weeks apart, to remind recipients to complete the survey or forward the survey to relevant, qualified personnels within the PHU based on their experiences during the COVID-19 pandemic and vaccine rollout. Reminder emails were terminated once the respondent completed or declined to complete the survey. A final follow-up was sent to respondents who declined to participate to solicit reasons for their decision. Survey collection was implemented between 1 June 2022 to 22 December 2022.

### Data Collection and Analysis

Study data were collected and managed using REDCap electronic data tools hosted at the University of Toronto [42, 43]. Descriptive analysis of responses was performed in Microsoft Excel.

## Results

The survey was distributed to 34 PHUs and 28 PHUs responded, yielding a response rate of 82.5%. The survey item completion rate was 100%.

### Respondent Characteristics and PHU Ethnoracial Priority Populations

Of the 28 respondents representing 28 PHUs, participants on average had 3 years of experience (range 1–30 years). See Table 1 for respondent role within their PHUs.

**TABLE 1:** Respondent characteristics (Ontario, Canada, 2024).

Respondent role within respective PHU	Number of respondents
Medical Officer of Health	4
Chief Nursing Officer	3
Director	10
Immunization program supervisor	2
Program manager	7
Public health nurse	1
Epidemiologist	1
Total	28

Perceived effectiveness of public health unit partnerships with faith-based and other community-based organizations to promote COVID-19 vaccination among ethnoracial communities, Ontario, Canada, 2022.

**FIGURE 1:**
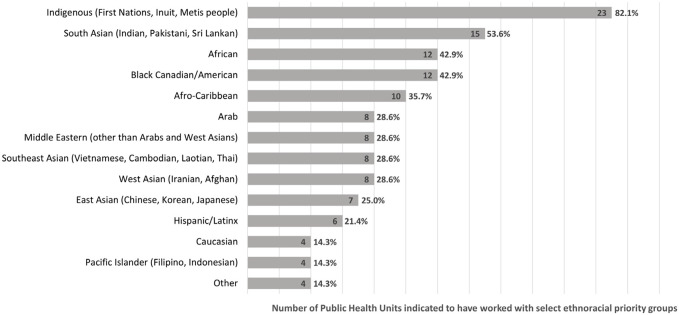
Priority ethnoracial communities during COVID-19 vaccine rollout (N = 28 public health units) (Ontario, Canada, 2024). Perceived effectiveness of public health unit partnerships with faith-based and other community-based organizations to promote COVID-19 vaccination among ethnoracial communities, Ontario, Canada, 2022.

PHUs identified 13 main ethnoracial communities that were prioritized for COVID-19 vaccine promotion in their area (see Figure 1). The majority of PHUs identified Indigenous and South Asian populations as priority communities (23/28, 82.1% and 15/28, 53.6% respectively); four (4/28, 14.3%) PHUs also identified Caucasian as a priority group, specifically those belonging to Anabaptist and Low German Speaking Mennonite communities as well as Ukrainian refugees.

### Partnerships to Deliver Ethnoracial, Including Faith-Based, Vaccine Interventions

**FIGURE 2:**
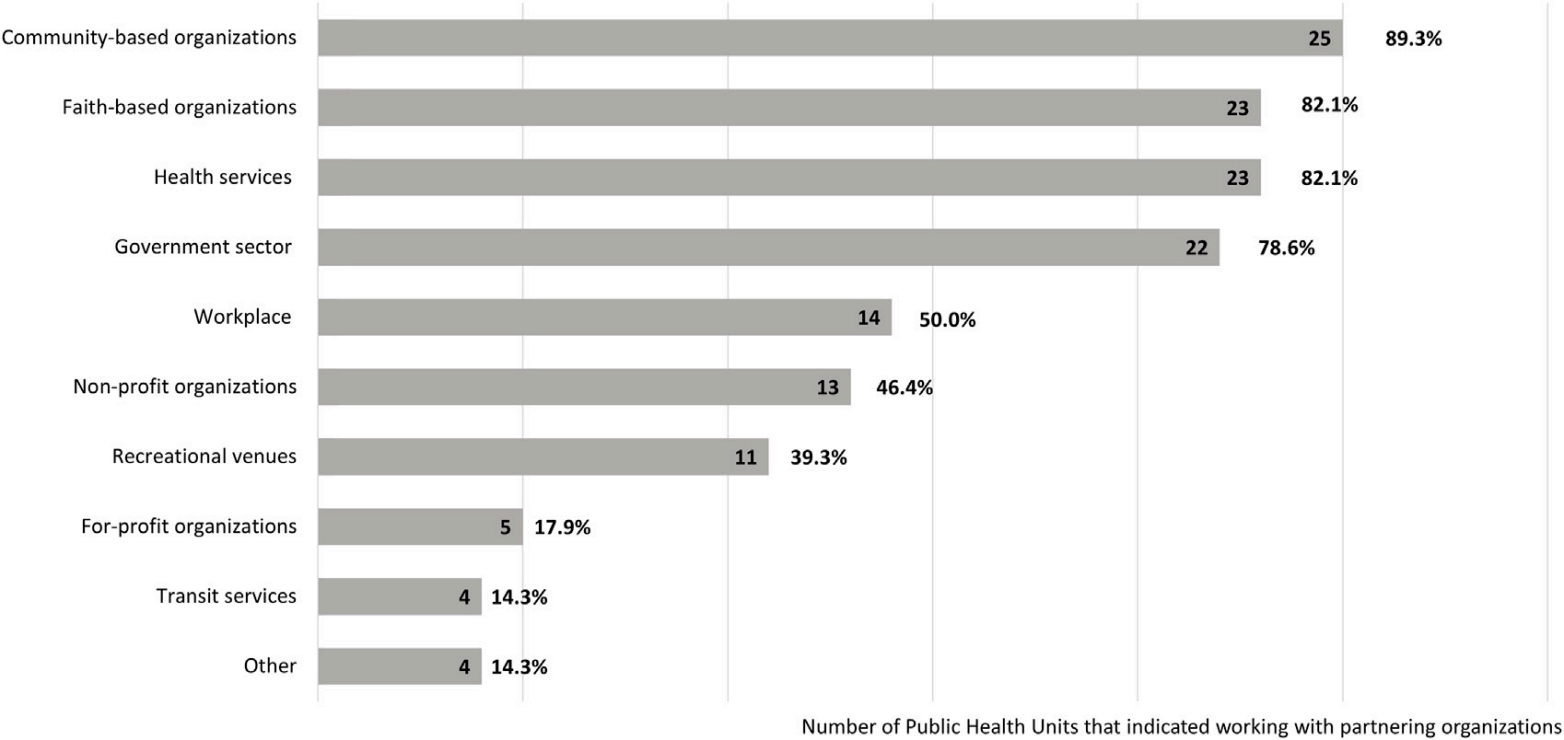
Key partners for vaccine rollout to ethnoracial and vulnerable populations (N = 28 public health units) (Ontario, Canada, 2024). Perceived effectiveness of public health unit partnerships with faith-based and other community-based organizations to promote COVID-19 vaccination among ethnoracial communities, Ontario, Canada, 2022.

PHU respondents listed several categories of partner organizations that supported vaccine rollout to ethnoracial communities (see Figure 2). Majority of PHUs partnered with CBOs (25/28, 89.3%), FBOs (23/28, 82.1%), health systems services and facilities (e.g., hospitals; 23/28, 82.1%), and government sector (e.g., provincial/municipal/regional services; 22/28, 78.6%). PHUs also partnered with workplaces (e.g., large factories); public and private recreational facilities (e.g., community centres); non-for-profit organizations (e.g., immigration services); for-profit organizations (e.g., pharmaceutical companies and businesses); and transit services (e.g., local busing systems). Four (4/28, 14.3%) PHUs indicated partnering with “other” organizations, which respondents identified as local First Nations Groups and local ethnocultural organizations (e.g., South Asian community groups, Latinx community groups, Black community organizations).

PHU respondents identified several methods to engage FBOs to support the vaccination program, with email and phone contact with FBOs as the starting point for most PHUs (60.7% email and 50.0% phone contact, respectively). Other examples of partnership initiation included word-of-mouth (14/28, 50.0%), such as through direct personal relationships with PHU staff. PHUs also sought to reach FBOs through print media (7/28, 25.0%), webpages hosting vaccine information for faith communities (6/28,21.4%), as well as tailored messaging on social media for faith-based communities (4/28, 14.3%). PHUs also invited faith leaders to vaccine forums and virtual townhalls to encourage their participation in the vaccine campaign, or where needed, provided in-person message delivery by public health nurses (e.g., to Mennonite communities and the Amish). In some instances, FBOs had initiated first contact with PHUs when, for example, offering their places of worship as vaccine clinics.

Three of the 6 PHUs involved in the High Priority Communities Strategy partnered extensively with FBOs to advance the strategy’s goals. They relied on churches (e.g., branches of Christian churches that deliver sermons in non-official Canadian languages to ethnic populations that primarily speak other languages), mosques, and faith-based community services to reach marginalized communities that often overlap with low-income, high material deprivation, lower socioeconomic status, and contain higher percentage of immigrants, newcomers, and seasonal workers whose mother tongue is non-English. Three PHUs reported partnering minimally with FBOs; one PHU indicated working with Indigenous communities to improve vaccine uptake by increasing accessibility and reducing barriers by addressing questions and concerns.

### Description of Vaccine Program Delivery

#### Social Media Strategies for Ethnoracial Communities

Social media strategies were instrumental to vaccination efforts, especially in the engagement of populations who do not speak English and faith communities. Over half (16/28, 57.1%) of the PHUs used dedicated social media strategies (i.e., reaching out, listening in) to promote vaccine rollout to reach priority populations in their respective jurisdictions. PHUs’ official Twitter, Facebook, Instagram, and YouTube were the most popular social media platforms. Some PHUs used WhatsApp messaging (4/28, 14.3%) to disseminate vaccine messaging to ethnoracial and faith-based communities. Vaccine promotional social media posts were translated into numerous languages (e.g., Hindu, Punjabi, Urdu, Spanish, and Mandarin) to reach ethnoracial populations that speak non-official Canadian languages, and cross-posted to amplify vaccine campaigns already in print and on news media. Some PHUs used geo-targeted features of Facebook and Instagram to target geographical “hotspots” defined by forward sortation area, based on higher COVID-19 infection rates where sizable ethnoracial groups reside and low vaccination rates.

For some PHUs, social media strategies were age-based and focused on reaching the most people within specific age groups. However, in some cases this included setting up vaccine clinics in universities and designing vaccine promotional messaging to encourage vaccination among marginalized ethnoracial international students.

Over forty percent (12/28, 43.0%) of PHUs did not have dedicated social media strategies to reach priority ethnoracial communities. Out of these 12 units, 6 indicated that this is because their priority ethnoracial groups did not use social media, while 6 others considered reaching out via social media as less effective than other approaches. In these areas, social services agencies, phone, print media, community clinics or primary care providers with existing working relationships with locals, were the primary means to reach priority populations.

#### Partnerships With FBOs

Most PHUs (23/28, 82.1%) reported partnership with FBOs and described FBO participation in PHU vaccine rollout intervention in at least 4 ways: organizing and lending space for pop-up clinics (e.g., mobile vaccine clinics, vaccine drives); facilitating the dissemination and design of faith-based vaccine educational information (e.g., townhalls, Q&As); community outreach (e.g., community ambassadors, door-to-door outreach); and promoting awareness through media campaigns (e.g., traditional and new media advertisement). Table 2 describes the range of FBO entities that PHUs engaged with according to the size of the populations that they serve. The table also describes the types of FBOs, and the different places of worship that PHUs engaged with during the vaccine rollout.

**TABLE 2:** Partnerships with faith-based organizations (Ontario, Canada, 2024).

Number of FBO partnerships based on size of PHU region		
Large-sized PHUs (population of more than 1 million people)	Count	(%)
Ottawa Public Health Peel Public Health Toronto Public Health ^a^York Region Public Health	67–150	
Mid-sized PHUs (population between 500–999 thousand)		
Durham Region Health Unit Hamilton Health Services Simcoe Muskoka District Health Unit Waterloo Public Health	20–60	
Small-sized PHUs (population between 100–499 thousand)		
Algoma Public Health Brant County Health Unit Eastern Ontario Health Unit Grey Bruce Health Unit Haldimand-Norfolk Health Unit Huron Perth Public Health Middlesex-London Health Unit North Bay Parry Sound District Health Unit Renfrew County and District Health Unit Kingston, Frontenac, Lennox & Addington Public Health ^b^Porcupine Health Unit Southwestern Public Health Timiskaming Health Unit Public Health Sudbury and Districts Wellington-Dufferin-Guelph Public Health	>10	
^c^ **Types of FBOs**		
Places of worship	16/23	69.6
Social service organizations	15/23	65.2
Educational Institutions	14/23	60.9
Community/leisure centre	8/23	34.8
Advocacy association	6/23	26.1
Charity	1/23	4.3
Other	2/23	8.7
Places of Worship		
Churches	19/23	82.6
Mosques	9/23	39.1
Temples	6/23	26.1
Gurdwaras	4/23	17.4
Synagogues	2/23	8.7

^a^ York Region Public Health only named 5 FBOs but indicated partnership with others.

^b^ Porcupine Health Unit has a population under 100,000.

^c^ Examples:

Faith-based social service organizations: Salvation Army, Good Shepherd, Young Women’s Christian Association.

Faith-based educational institutions: parochial schools and daycare centres.

Faith-based community/leisure centres: Five Oaks.

Faith-based advocacy associations: World Sikh Organization of Canada.

Faith-based charities: Canadian Centre for Christian Charities, Tzu-Chi Buddhist Society.

Perceived effectiveness of public health unit partnerships with faith-based and other community-based organizations to promote COVID-19 vaccination among ethnoracial communities, Ontario, Canada, 2022.

PHUs in large cities that serve geographical areas with larger and diverse ethnoracial populations partnered with faith-based entities (i.e., Christian, Islamic, Sikh, Hindu, Buddhist, and Judaism). Small-sized PHUs serving smaller regions or those with dispersed populations, focused on approaching specific FBOs such as the Mennonite and Catholic churches. PHUs with primarily rural populations with Indigenous communities, partnered primarily with Indigenous Urban Centres and Indigenous health/social services centres (e.g., Porcupine Public Health, Algoma Public Health, Peterborough Public Health; see Table 2).

Of the 23 PHUs that worked with FBOs, the most common partners were places of worship (16/23, 69.6%), faith-based social service institutions (15/23, 65.2%), and faith-based educational institutions (14/23, 60.9%). All 28 PHUs worked with local Catholic District School Boards, though five PHUs did not consider these school boards as FBOs. Other types of FBOs included faith-based community/leisure centres, advocacy associations and charities (see Table 2).

Five (5/28, 17.9%) PHUs did not use places of worship as sites for vaccine clinics; rather they utilized Indigenous sacred places, parochial schools, Mennonite community services, community newspaper and radio stations.

### PHU Self-Assessment of the Interventions

**FIGURE 3:**
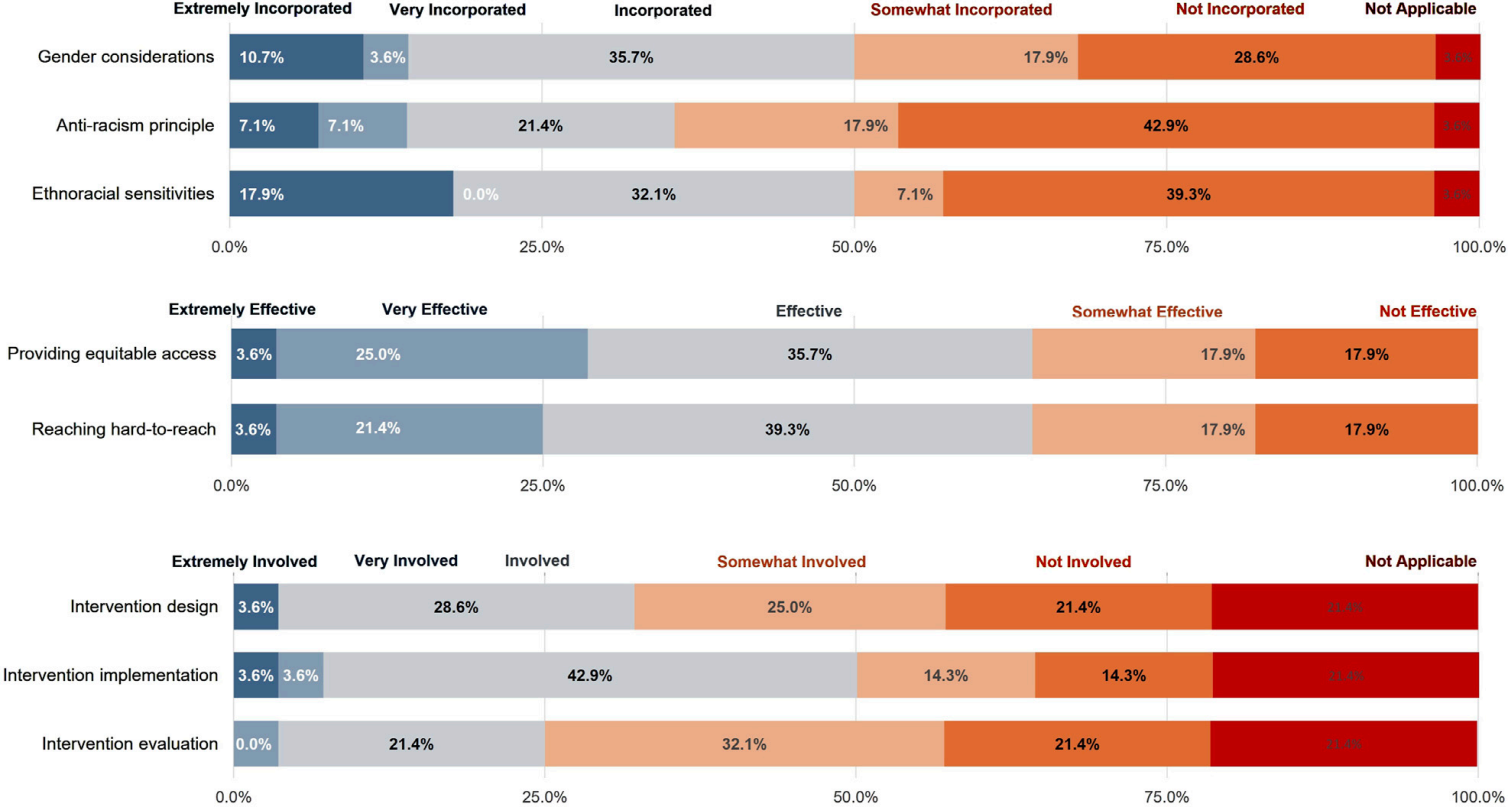
Extent that faith-based vaccine interventions incorporated anti-racism principles, ethnoracial sensitivities, and gender considerations **(A)**; Extent that faith-based organizations were effective in advancing equitable access and reaching hard-to-reach populations **(B)**; Extent that faith-based organizations are involved in the design, implementation, and evaluation of vaccine interventions **(C)** (Ontario, Canada, 2024). Perceived effectiveness of public health unit partnerships with faith-based and other community-based organizations to promote COVID-19 vaccination among ethnoracial communities, Ontario, Canada, 2022.

On a 5-point Likert Scale, 10 (10/28, 35.7%) PHUs considered faith-based vaccine delivery programs to have “extremely incorporated” gender considerations, while 8 (8/28, 28.6%) considered them not to have incorporated these considerations (See Figure 3). More than half (15/28, 53.5% and 16/28, 57.1%) incorporated some degree of anti-racism principles and ethnoracial sensitivities, respectively.

Majority of PHUs (23/28, 82.1%) considered FBO partnerships as effective in increasing vaccine uptake among hard-to-reach populations and providing equitable access (See Figure 3). These PHUs scored an average of 4.16 (Effective) out of 5 (Very Effective): Middlesex London, Toronto Public Health, Ottawa Public Health, City of Hamilton Health Services, Peel Public Health.

Five PHUs (5/28, 17.9%) considered FBO partnerships to be ineffective in reaching hard-to-reach populations and providing equitable access. These PHUs scored an average of 1 (Not Effective) out of 5 (Very Effective): Niagara Region Public Health, Lambton Public Health, Algoma Public Health, Kingston, Frontenac and Lennox & Addington Public Health and Sudbury & District Health Unit. Three indicated that they did not engage with FBOs in their vaccine rollout plan.

Reasons for an ineffective rating included not having sizeable hard-to-reach populations within their jurisdiction or receiving lukewarm responses from FBOs and therefore not pursuing a partnership. Other PHUs expressed that other community outreach and equity-based interventions were more effective than working directly with FBOs. For instance, some PHUs relied on CBOs with strong connections to ethnic communities with faith affiliations to promote vaccines, and found faith-based programs to be less effective at reaching out to diverse ethnoracial populations.

**FIGURE 4:**
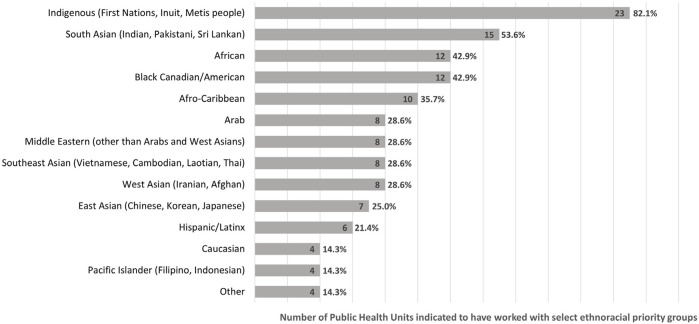
Evaluation methods to measure faith-based interventions for vaccine uptake (N = 28 public health units) (Ontario, Canada, 2024). Perceived effectiveness of public health unit partnerships with faith-based and other community-based organizations to promote COVID-19 vaccination among ethnoracial communities, Ontario, Canada, 2022.

Twelve (12/28, 42.9%) PHUs reported use of formal and informal methods to evaluate vaccine intervention success of faith-based and community partnerships (See Figure 4). Four (4/28, 14.3%) PHUs reported using formal evaluation methods such as surveys with CBO/FBOs, interviews with key informants in the community, and focus groups with target populations and faith-based partners. Eight (8/28, 28.6%) PHUs reported using informal evaluation, such as verbal and written feedback from faith leaders and community members, post-event/community outreach/vaccine clinic debriefs with attendees, and *ad hoc* surveys/questionnaires with community leaders and FBO/CBO partners.

Twelve (12/28, 42.9%) PHUs reported not to have relied on any formal or informal evaluation methods. Among these, 4 (4/28, 14.3%) PHUs reportedly relied on monitoring changes in vaccine uptake in areas with high COVID-19 infection and low vaccine rates. The data were combined with local knowledge on ethnoracial populations within PHUs through existing public health programs (e.g., “School Immunization Program”, “Healthy Babies Healthy Children” program, “EarlyON”), and that of partners (e.g., other public sectors, local community health centres) to determine intervention success.

## Discussion

The uptake of vaccine interventions is influenced by social, cultural, and religious norms. Ethnoracial communities made structurally vulnerable may be hesitant because they have experienced systemic racism and been historically marginalized [44, 45]. In Ontario, 16.3% of the population reports being affiliated with a non-Christian religion, the highest proportion in Canada. As such, against the backdrop of inequalities exacerbated by a global health emergency like the COVID-19 pandemic [12], partnerships between public health organizations and FBOs were one among a suite of important strategies to promote vaccination among communities made structurally vulnerable. These partnerships hold potential for addressing other vaccine intervention priorities, but ongoing monitoring and evaluation is needed to measure their impact and to mitigate any potential unintended consequences.

In our study, the majority of PHUs expressed a desire to engage local FBOs in their COVID-19 vaccination initiatives during the pandemic. In PHU jurisdictions with diverse ethnocultural groups, faith-based partnerships were perceived as necessary and effective partners in improving vaccine uptake among ethno-racial groups made structurally vulnerable. These PHUs were more likely to incorporate gender and race-based concerns of these groups in intervention design and implementation, compared to PHUs in jurisdictions serving less diverse ethnocultural groups. While we found that faith-based initiatives were perceived as effective in delivering equitable vaccine distribution and uptake, the majority of PHUs we surveyed were not able to implement formal evaluation approaches to assess the impact of faith-based outreach efforts.

Interventions to boost vaccine uptake and epidemiological surveillance on the impact of interventions on groups made structurally vulnerable would benefit from applying a more uniform evaluation framework. The framework could include predetermined indicators and measurable outcomes to evaluate and improve programmatic delivery across the 34 PHUs and across Ontario. At the same time, local contextual and demographic factors should be incorporated into intervention design before implementation, and evaluation of vaccine uptake. As an example, programs like the Interfaith Health Program (IHP) led by Emory University collaborate with hundreds of FBOs across 10 public health sites across the United States [38, 46]. The program developed a relational model involving foundational beliefs, processes, and infrastructure called the “Model Practices Framework.” The model resulted from a modified Delphi technique which identified key faith-based locations for reaching vulnerable and marginalized populations after analyzing and synthesizing 4 years of progress reports and presentations [38, 46]. A unifying framework of best practice for PHUs ought to be useful in this context.

Although alliances between FBOs and public health are quite well-documented through programs including public health agency missions, congregation-based health promotion and disease prevention, and community outreach to priority populations, these efforts have received limited attention in evaluation efforts [47]. Our study reflected the lack of evaluation efforts observed in the broader literature. Possible reasons for limited evaluations conducted by PHUs range from human and financial resource capacity constraints and a higher priority placed on getting vaccines in arms to a lack of an overall implementation framework that incorporates monitoring and evaluation of community-based outreach and no time for formal ethics application to conduct evaluations in real-time. Evaluation efforts are critical and essential for preventative public health program planning; questions like “*what works and what does not work, for whom and under what circumstances,”* are often left unanswered [47]. Our study results echo Levin’s observation and shed light on the realities of the limitations faced by PHUs related to lack of individual level race-based and other sociodemographic data [48], and the need to rely on neighbourhood level characteristics. These issues often led to misrepresentation of area income level [49], and constraints around documenting the vaccine status of migrant and undocumented populations [50]. Addressing gaps in evaluation of outcomes and community partnerships may help to develop best practices to strengthen and sustain community engagement post-pandemic [10].

### Strengths and Limitations

Our study has several strengths. The survey addressed an understudied topic in Canada of PHU-FBO partnerships and used robust survey development methods that captured partnership processes and interventions involving PHUs and FBOs during a global health emergency. The study also had a strong response rate which covered a wide range of PHUs in terms of geographical region, size of jurisdiction and ethnoracial diversity of populations served.

We note some limitations in our study. The limited sample size precludes any statistical tests. Given the cross-sectional design, we were not able to assess the quality of partnerships or the growth in partnership over time. In our data collection, we also noted differences in interpretation of what was considered faith based. For example, some respondents did not consider engagement with Indigenous groups as faith-based, whereas some interpret spiritual engagement as faith-based. This was addressed in the interpretation of our results where possible.

### Conclusion

FBOs can be a valuable community partner as PHUs work to increase reach of health messages and uptake of public health interventions. Future work should focus on data availability particularly timely, nimble, and ethical data collection and disaggregating data on the basis of race, gender and other social stratifiers to inform more nuanced analyses. PHUs with sizeable ethnoracial diversity could consider facilitating knowledge exchange about promising implementation guidelines amongst PHUs with contextual and demographic similarities. Finally, there should be consideration for strengthening the capacity of PHUs to engage with CBOs/FBOs and measure implementation processes and outcome evaluations. Ongoing monitoring and evaluation of the impact of FBO engagement on vaccine confidence and uptake among ethnoracial communities is needed to inform future community engaged vaccine programming in Ontario and other jurisdictions.

## Data Availability

The anonymized survey responses for the 28 PHUs are available upon request.
