# Supplementary material for: Perceived Effectiveness of Public Health Unit Partnerships With Faith-Based and Other Community-Based Organizations to Promote COVID-19 Vaccination Among Ethnoracial Communities
Source: Int J Public Health. 2024 Nov 12;69:1607200. doi: 10.3389/ijph.2024.1607200 (PMC11626644; doi:10.3389/ijph.2024.1607200)
Supplement: Supplementary file 1 [file DataSheet1.PDF]

Improving vaccine confidence among minoritized communities: Survey Questions

---

Please check "Yes" if you agree to participate in this survey to proceed.

- Yes, I consent and understand.

Please select the Public Health Unit (PHU) you work for in the drop-down menu (list all 34 PHUs in drop down menu).

- Algoma Public Health
- Brant County Health Unit
- Chatham-Kent Public Health
- Durham Region Health Department
- Eastern Ontario Health Unit
- Grey Bruce Health Unit
- Haldimand-Norfolk Health Unit
- Kawartha, Pine Ridge District Health Unit
- Halton Region Health Department
- City of Hamilton Public Health Services
- Hastings Prince Edward Public Health
- Huron Perth Public Health
- Frontenac, Lennox & Addington Public Health
- Lambton Public Health
- Grenville and Lanark District Health Unit
- Middlesex-London Health Unit
- Niagara Region Public Health
- North Bay Parry Sound District Health Unit
- Northwestern Health Unit
- Ottawa Public Health
- Peel Public Health
- Peterborough Public Health
- Porcupine Health Unit
- Public Health Sudbury & Districts
- Renfrew County & District Health Unit
- Simcoe Muskoka District Health Unit
- Southwestern Public Health
- Thunder Bay District Health Unit
- Timiskaming Health Unit
- Toronto Public Health
- Region of Waterloo, Public Health
- Wellington-Dufferin-Guelph Public Health
- Windsor-Essex County Health Unit
- York Region Public Health

(Source: https://www.health.gov.on.ca/en/common/system/services/phu/locations.aspx)

What is your role/position at the [phu]? if you prefer not to say, please type "prefer not to say".
____________________________________ (e.g., managing director, COVID-19 response)

How many years have you been involved in immunization programs?
___________________________________ (If less than one year of experience, type 1.)

Part 1. Working with minoritized populations during COVID-19 (Part 1 of 5)

What are the ethno-racial origins of the population that [phu] serve? Please select all that apply.

- o African
- o Afro-Caribbean
- o Arab
- o Central European (Italian, Greek)
- o East Asian (Chinese, Korean, Japanese)
- o Eastern European (Ukrainian, Russian)
- o Indigenous (First Nations, Inuit, Metis people)
- o Latinx
- o Middle Eastern (other than Arabs and West Asians)
- o Pacific Islander (Filipino, Indonesian)
- o South Asian (Indian, Pakistani, Sri Lankan)
- o Southeast Asian (Vietnamese, Cambodian, Laotian, Thai)
- o West Asian (Iranian, Afghan)
- o Western European (English, Scottish, Irish,
- o French, German, Dutch)
- o Other group

If selected other group, please specify: ___________________________________

Please select all ethno-racial communities that the COVID-19 vaccine intervention target:

- o African
- o Afro-Caribbean
- o Arab
- o Black Canadian/American
- o East Asian (Chinese, Korean, Japanese)
- o Indigenous (First Nations, Inuit, Metis people)
- o Latinx
- o Middle Eastern (other than Arabs and West Asians)
- o Pacific Islander (Filipino, Indonesian)
- o South Asian (Indian, Pakistani, Sri Lankan)
- o Southeast Asian (Vietnamese, Cambodian, Laotian, Thai)
- o West Asian (Iranian, Afghan)
- o Other group

If selected other ethno-racial communities, please specify: ___________________________________

Which vaccine promotion intervention(s) have been implemented by [phu] throughout the COVID-19 pandemic to encourage immunizations among ethno-racial communities? Please select all that apply.

- Identifying or targeting risk groups (e.g., conducting surveys and interviews to understand knowledge, willingness to receive vaccination, and willingness to participate in vaccine promotion and vaccine concerns)
- Online vaccine awareness (Digital/virtual materials to inform the public where vaccinations are available and when, e.g., social media postings, webinars, podcasts)
- In-person vaccine awareness (In-person promotional materials to provide information where vaccinations are available and when, e.g., flyers, posters, media campaign)
- Online education sessions (Online dissemination to provide information on vaccine safety, vaccination eligibility, and to address vaccination concerns, e.g., online materials, Q&A sessions, virtual town hall meetings)
- In-person education sessions (In-person dissemination to provide information on vaccine safety, vaccination eligibility, and to address vaccination concerns e.g., home visits, in-person consultations)
- Online outreach to promote vaccination (Digital/virtual outreach to remind the public to get vaccinated, e.g., text, messaging, telephone calls)
- In-person outreach to promote vaccination (In-person outreach to remind the public to get vaccinated, e.g., door-to-door canvassing, community ambassador events, in-home visits)
- Targeted vaccination delivery (In-person, e.g., vaccination clinic, vaccine events, mobile vaccine clinic)
- Other services (Additional referral to address other needs, e.g., medical, security, and social services)
- Allied professional training (education and knowledge sharing for faith-based organizations/ community-based organizations, e.g., a symposium on health, leadership workshop, conferences to discuss community mobilization)
- Other

If selected "other", please describe what interventions took place to promote vaccination among ethno-racial communities. ____

Did you use a dedicated social media strategy to increase vaccine uptake among ethno-racial communities?

- Yes
- No

Describe [phu]'s dedicated social media strategy to increase vaccine uptake among ethno-racial communities. ____

What age group(s) did the intervention(s) to promote COVID-19 vaccines target among ethno-racial communities? Select all that apply.

- Seniors (65+)
- Adults (18+)
- Adolescents (12-17)
- Children (5-11)

What organizations are primarily involved in your interventions to promote the uptake of COVID-19 vaccines among ethno-racial communities? Please select the top 5 that apply.

- Community-based organizations
- Faith-based organizations
- For-profit organizations (e.g., private companies, pharma, paramedic companies)
- Government sector (e.g., provincial/municipality/city services, school boards)
- Healthcare setting (hospitals, CHC, etc)
- Non-profit organizations
- Recreation (e.g., community centres)
- Transit services
- Workplace (e.g., schools, employment location)
- No partner organizations were involved
- Other

If chosen "other", please insert other organizations/agencies involved in the COVID-19 vaccine uptake intervention among ethno-racial communities: ____

As a reference, the image illustrates a sample of collaborators in a local vaccination task force.

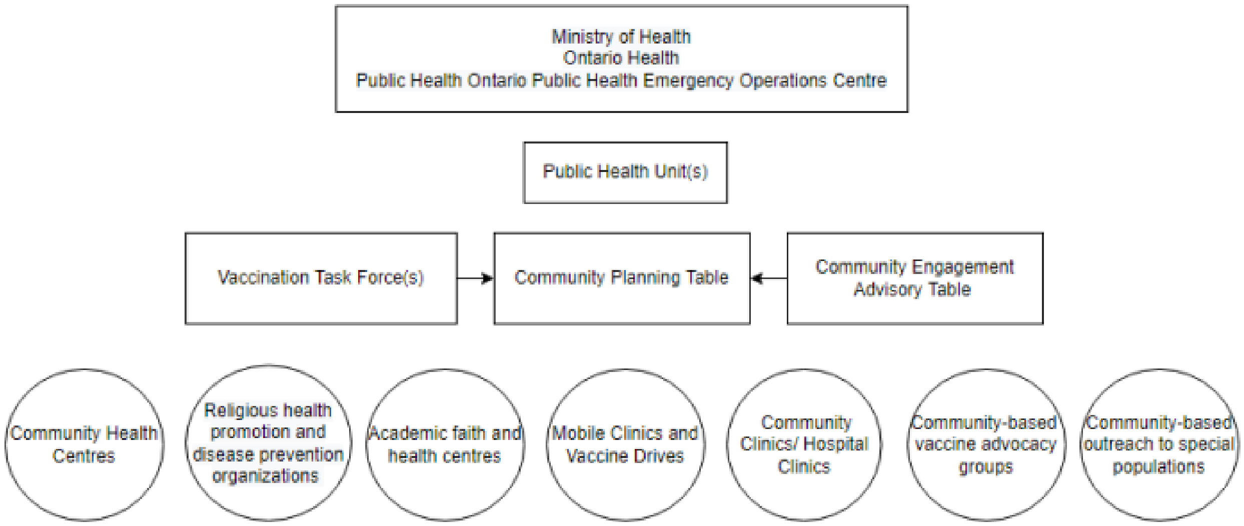

Part 2. Participation in Ontario's High Priority Community Strategy (Part 2 of 5)

Is your PHU involved in the High Priority Community (HPC) strategy?
- Yes
- No

Which community neighborhoods does [phu] serve in the HPC strategy?

____

Please select priority population groups in your service area. Please select all that apply:
- Hard-to-reach populations (rural, persons with substance use, victims of violence, homeless)
- Racial and linguistic minority groups
- Newcomers and Immigrants
- Persons with disabilities
- Persons requiring social assistance

- Low-income populations
- Seniors
- Known vaccine hesitant groups/communities
- Other

If selected "other" population, please specify: ____

Is [phu] collaborating with faith-based organizations and/or faith leaders in the HPC Strategy?

- Yes
- No

Please specify all the community-based and faith-based partners involved in the HPC strategy. if not applicable, please input "N/A". ____

Part 3. Faith-based and community collaborations (Part 3 of 5)

What are the outreach methods used to recruit faith leaders and faith-based organizations? Select all that apply:

- Email
- Telephone
- Physical poster/flyer
- Social media
- Website
- Word-of-mouth
- Not applicable, did not involve faith-based groups or religious leaders
- Other

If "other", what are the outreach methods used to recruit faith leaders and faith-based organizations? ____

Which of the interventions to promote the uptake of COVID-19 vaccines involve faith-based organizations? Please select all that apply.

- Awareness raising campaigns
- Community outreach strategies (e.g., community ambassadors, door-to-door outreach, ads in newspapers)
- Educational information provision
- Pop-up community vaccine clinics/ mobile vaccine clinics (e.g., vaccine drive in place of worship or community health centre)
- None of the above
- Other

If selected "other", please specify". ____

Which faith-based organizations are involved in the intervention(s) to promote the uptake of COVID-19 vaccines? Please select all that apply.

- Places of worship (e.g., churches, mosques, synagogues, temples, gurdwaras)

- Faith-based community/leisure centres (e.g., Young Women's Christian Association, Christian Life Centre, Five Oaks)
- Faith-based social service organizations (e.g., Salvation Army, Good Shepherd)
- Faith-based educational institutions (e.g., faith-based schools, daycares)
- Faith-based advocacy associations (e.g., World Sikh Organization of Canada)
- Faith-based charity (e.g., Canadian Centre for Christian Charities, Tzu-Chi Buddhist Society)
- Not applicable, did not involve faith-based organizations
- Other

If selected "other" faith-based organizations, please specify:

____

Please list all faith-based organizations, religious entities, and/or religious leaders (URLs allowed) that have participated in improving vaccine uptake in your PHU.

____

If [phu] is working with faith-based organizations that include places of worship, please select which ones:

- Churches
- Gurdwaras
- Temples
- Mosques
- Synagogues
- Other

Please specify the places of worship if selected "other".

____

Part 4. Evaluating COVID-19 vaccine uptake interventions (Part 4 of 5)

To what extent are faith leaders and/or faith-based organizations involved in the design of [phu]'s intervention(s) to promote the uptake of COVID-19 vaccines?

Not involved — Involved — Extremely involved

(Place a mark on the scale above)

To what extent are faith leaders and/or faith-based organizations involved in the implementation of [phu]'s intervention(s) to promote the uptake of COVID-19 vaccines?

Not involved — Involved — Extremely involved

(Place a mark on the scale above)

To what extent are faith leaders and/or faith-based organizations involved in the evaluation of [phu]'s intervention(s) to promote the uptake of COVID-19 vaccines?

Not involved	Involved	Extremely involved

(Place a mark on the scale above)

To what extent are intervention(s) developed by/with faith-based organizations promoting COVID-19 vaccine tailored to ethno-racial minorities?

Not applicable to racialized minorities	Applicable to racialized minorities	Extremely applicable to racialized minorities

(Place a mark on the scale above)

To what extent are intervention(s) developed by/with faith-based organizations to promote COVID-19 vaccines co-created with anti-racism principles?

Not applicable to racialized minorities	Applicable to racialized minorities	Extremely applicable to racialized minorities

(Place a mark on the scale above)

To what extent is gender considered in the design of the intervention(s) involving faith-based organizations to promote the uptake of COVID-19 vaccines?

Gendered concerns not incorporated	Gendered concerns incorporated	Gendered concerns extremely incorporated

(Place a mark on the scale above)

How effective do you think the intervention(s) of your PHU that involve faith leaders and/or faith-based organizations to promote the uptake of COVID-19 vaccines in reaching hard-to-reach populations (e.g. ethnic minorities, religious minorities, gendered minorities)?

Not effective	Effective	Extremely effective

(Place a mark on the scale above)

How effective are intervention(s) that involve faith leaders and/or faith-based organizations in promoting equitable access to COVID-19 vaccines?

Not effective	Effective	Extremely effective

(Place a mark on the scale above)

Are you using formal evaluation methods to measure intervention success of faith-based and community-based partnerships?

- ○ Yes
- ○ No

Please specify formal and informal evaluation methods and tools used to measure intervention success. Please share URLs here if any. If none, please indicate N/A.

____

Part 5. Other information we should learn about [phu]'s vaccine uptake efforts (Part 5 of 5)

Please paste any URLs (e.g., COVID-19 vaccine roll-out plan; promotional material used to promote COVID-19 vaccines) that you think may help us further understand your PHU's work to increase COVID-19 vaccine uptake. ____

Please upload one file (e.g., COVID-19 vaccine roll-out plan; promotional material used to promote COVID-19 vaccines) that you think may help us further understand your PHU's work to increase vaccine uptake among ethno-racial communities. File size is limited to 30MB.

Please upload a second file (e.g., COVID-19 vaccine roll-out plan; promotional material used to promote COVID-19 vaccines) that you think may help us further understand your PHU's work to increase vaccine uptake among ethno-racial communities. File size is limited to 30MB.

We would really appreciate the opportunity to interview you to learn about the many ways in which you and your PHU support minoritized populations to get vaccinated over the past 2 years during COVID-19. Your contribution will allow us to understand how PHUs in Ontario work with faith-based organizations and community-based organizations to improve vaccine uptake. Please check yes if you agree to be contacted by email following this survey, thank you.

- ○ Yes
- ○ No

Your email ____________________________________ (Please insert an email where we can contact you).

Your phone ____________________________________ (Please insert a phone number where we can contact you.)

Thank you for participating in this survey. Please indicate how you would like to be acknowledged as a participant of the survey should we publish the survey results.

- ○ I would like to be acknowledged by my first name and job title at my PHU (e.g., survey participant: (first name), Director of COVID-19 immunization operations at [phu])
- ○ I would like to be acknowledged by my job title at my PHU (e.g., survey participant: Director of COVID-19 immunization operations at [phu])
- ○ I would like to be acknowledge by the PHU only, please leave my first name and job title out. (e.g., participating PHU: [phu])
- ○ I prefer not to be acknowledged.

First Name ____________________________________ (Leave blank if want to remain anonymous)

Last Name ____________________________________ (Leave blank if want to remain anonymous)

Thank you [first_name], you have reached the end of the survey. Your contribution is extremely important to us. Please consent to submitting your survey results.

- o I consent to submitting my survey
